# The Eye, Oxidative Damage and Polyunsaturated Fatty Acids

**DOI:** 10.3390/nu10060668

**Published:** 2018-05-24

**Authors:** Sergio Claudio Saccà, Carlo Alberto Cutolo, Daniele Ferrari, Paolo Corazza, Carlo Enrico Traverso

**Affiliations:** 1 Ophthalmology Unit, IRCCS Ospedale Policlinico San Martino, Viale Benedetto XV, 16132 Genoa, Italy; ferraridaniele92@gmail.com; 2 Clinica Oculistica, DiNOGMI, University of Genoa and IRCCS Ospedale Policlinico San Martino, Viale Benedetto XV, 16132 Genoa, Italy; cacutolo@gmail.com (C.A.C.); polcorazza@gmail.com (P.C.); mc8620@mclink.it (C.E.T.)

**Keywords:** oxidative damage, primary open angle glaucoma, GSTM1, POAG, glaucoma epidemiology

## Abstract

Polyunsaturated fatty acids (PUFA) are known to have numerous beneficial effects, owing to their anti-inflammatory and antioxidant properties. From a metabolic standpoint, the mitochondria play a fundamental role in cellular homeostasis, and oxidative stress can affect their functioning. Indeed, the mitochondria are the main source of ROS, and an imbalance between ROS and antioxidant defenses leads to oxidative stress. In addition, aging, the decline of cellular functions, and continual exposure to light underlie many diseases, particularly those of the eye. Long-term exposure to insults, such as UV light, visible light, ionizing radiation, chemotherapeutics, and environmental toxins, contribute to oxidative damage in ocular tissues and expose the aging eye to considerable risk of pathological consequences of oxidative stress. Ample antioxidant defenses responsible for scavenging free radicals are essential for redox homeostasis in the eye, indeed, eye tissues, starting from the tear film, which normally are exposed to high oxygen levels, have strong antioxidant defenses that are efficient for protecting against ROS-related injuries. On the contrary, instead, the trabecular meshwork is not directly exposed to light and its endothelial cells are poorly equipped with antioxidant defenses. All this makes the eye a target organ of oxidative damage. This review focuses on the role of the polyunsaturated fatty acids in the human eye, particularly in such pathologies as dry eye, glaucoma, and macular degeneration, in which dietary PUFA supplementation can be a valid therapeutic aid.

## 1. Introduction

Omega-3 polyunsaturated fatty acids (PUFA) are essential to the proper functioning of the human organism, as they are fundamental elements of neurons, nerve terminals, cell membranes, and myelin. Omega-3 docosahexaenoic acid (DHA) is essential for the proper functioning [1] and development of the brain [2]. Furthermore, it is the main omega-3 polyunsaturated fatty acid present in the brain [3] PUFA contribute to the physical-chemical properties of the cell membrane by influencing membrane permeability and, therefore, the transport functions and ion channels. Their enzymatic functions and cellular receptors, whose functions are based on the fluidity of the cell membrane, depend on the number and type of fatty acids. Findings indicate that, thanks to the receptors, PUFA are able to regulate signaling processes and, consequently, influence gene expression [4]. DHA can be obtained directly from the diet or synthesized in the body from α-linolenic acid [5]. They are preferentially incorporated as structural components into membrane phospholipids [6]. DHA is the main fatty acid of the central nervous system; indeed, it is an integral component of the phospholipids of the neural membrane [7] and is localized in the synaptosomes. Interestingly, in the disc membrane of retinal photo receptors, DHA contributes 50% of the total fatty acid content of the phospholipids and accounts for 75–100% of the fatty acids [8]. It can, therefore, influence neurogenesis, neuroplasticity, neurite growth, synaptogenesis, and membrane fluidity. Moreover, it improves the speed of signal transduction and neurotransmission and improves cognitive function [9]. These functions are mediated by N-Docosahexaenoylethanolamine (synaptamide), which is an endocannabinoid-like metabolite endogenously synthesized from DHA in the brain and retina [10]. Synaptamide may also be a mediator of the anti-inflammatory effect of DHA [3]. Actually, it is known that DHA protects against neuroinflammation [11], probably because it produces an increase in cAMP in dendritic cells, thereby reducing the production of proinflammatory mediators, such as TNF-α, IL-17, and IFN-γ, and increasing the production of anti-inflammatory factors, such as IL-10 [12]. 

## 2. Fatty Acids and Mitochondria

These signals can then influence the elaboration of mitochondrial energy, improving mitochondrial function [13], thereby influencing several aspects of cellular energy metabolism and neuronal plasticity. Metabolic activity can, in turn, also influence membrane homeostasis, which is able to support synaptic plasticity [14] and integrity and improve synaptic function [15]. Many diseases show a clear relationship between inflammation and oxidative stress [16]. At the cellular level, these processes lead to stress of the endoplasmic reticulum, to which the mitochondria are linked [17]; the mitochondria, in turn, show a dysfunction that activates the unfolded protein response (UPR). In turn, the UPR activates the main inflammatory pathways. Interestingly, this pathogenic mechanism occurs both in glaucoma [18] and in insulin resistance [19]. The excessive production of ROS induces peroxidation of the lipids in the cell membrane and the release of aldehydes, which damage the cells. The interaction between the homeostasis of energy in the neurons and synaptic plasticity is crucial to the maintenance of neuronal functioning as a whole and, hence, to neurological health. Finally, dietary PUFA are also known to influence mitochondrial calcium transport [20]. All of this not only underlies oxidative damage to the tissues, but also becomes very important to the eye. The aim of this review is to evaluate the possible role played by PUFA in counteracting oxidative damage, particularly in the main diseases of the eye.

## 3. Light and Oxidative Damage

**Figure 1:**
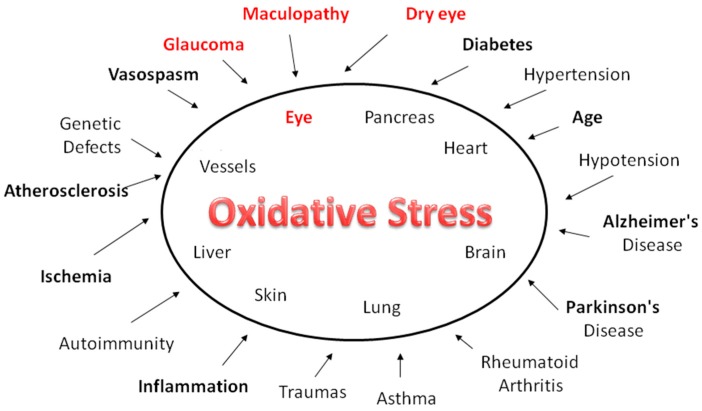
The human eye is constantly exposed to sunlight and artificial light. Exogenous sources of ROS, such as UV rays, visible light, ionizing radiations, chemotherapeutics, and environmental toxins, contribute to oxidative damage in the ocular tissues. In the long-term, such injuries expose the aged eye to considerable risk of the pathological consequences of oxidative stress. While this affects practically all organs and apparatuses, it has a heavy impact in diseases such as glaucoma, dry eye and age-related macular degeneration.

**Figure 2:**
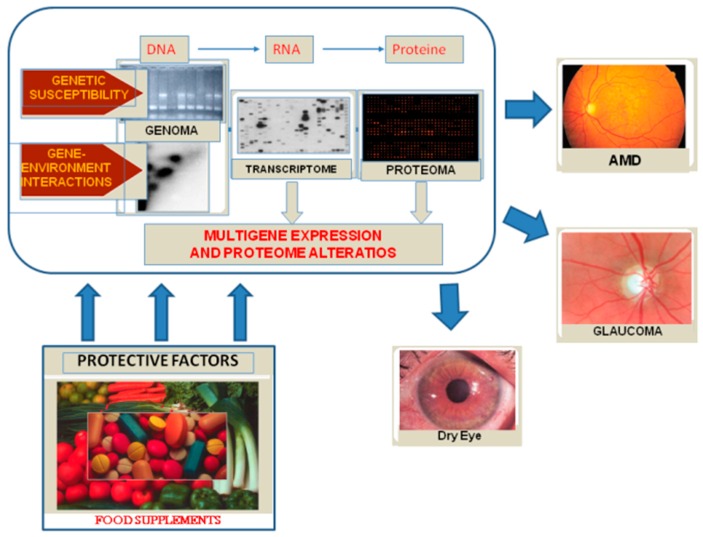
By interacting with genetic and environmental factors, oxidative stress results in DNA damage, alterations of gene expression and of the protein profile and, finally, disease.

The human eye is constantly exposed to sunlight and artificial light. These, and other exogenous sources of reactive oxygen species (ROS), such as ionizing radiation, chemotherapeutics, and environmental toxins, contribute to oxidative damage in the ocular tissues (Figure 1). Long-term exposure to these sources of injury constitutes a considerable risk for the aging eye, owing to the pathological consequences of oxidative stress. When light enters the eye, it gives rise to the formation of ROS, which must be kept in constant equilibrium with the cellular antioxidant defenses; if this balance is altered, oxidative stress occurs. Adequate levels of antioxidant defenses are, therefore, essential to redox homeostasis. Changes in ROS concentration influence the scavenging capacity of the cells. The amount of ROS in the tissues is normally relatively low. However, an increase in the levels of superoxide or nitric oxide leads to a temporary imbalance, which impair redox regulation. The persistent production of large quantities of ROS and RNS may lead to persistent changes in transduction signaling and gene expression, inducing pathological conditions. Oxidative and nitroxidative stress causes molecular damage to nucleic acids, lipids, and proteins, which can alter or compromise cell metabolism and viability, even inducing necrosis or apoptosis [21]. Over time, the ocular tissues, from the lacrimal film to the retina, suffer from oxidative stress; consequently, the antioxidant defenses of each tissue play an important role in safeguarding against degenerative eye diseases (Figure 2). The cornea and its epithelial cells are exposed to high levels of oxygen; however, they have a high antioxidant power and effective defenses against ROS injury. By contrast, structures, such as the trabecular meshwork (TM), which are not directly struck by light, are particularly sensitive to this type of damage [22]. Thus, environmental mutagens and endogenous sources of ROS play a fundamental pathogenic role in degenerative diseases of the eye. Nutrition modulates epigenetic markers, which have been linked both to increased risk of disease and to protection against disease. Moreover, appropriate nutrition can protect against pollution-induced inflammation through the epigenetic regulation of the pro-inflammatory target genes of the NF-KB pathway [23].

## 4. Oxidative Damage and Aging

Aging is characterized by a progressive decline in physiological functions and increased susceptibility to disease. This condition, which affects all living beings, can be explained through the “theory of free radicals”. According to this theory, aging and age-related diseases are the result of damage due to the free radicals produced by an imbalance between their formation and the antioxidant defenses [24] (Figure 1). The free radicals (ROS) produced during aerobic respiration have deleterious effects on the components of the cells and connective tissues; over time, the damage accumulates, leading to aging and death [25]. Moreover, it has been suggested that a central role in this process is played by the mitochondria, which generate a significant amount of cellular energy through the consumption of most of the intracellular oxygen. Indeed, about 90% of cellular oxygen is consumed inside the mitochondria, especially in the internal membrane, where oxidative phosphorylation occurs. In the long term, oxidative damage induces mitochondrial stress (deletion), which, over time, causes damage; the damaged mitochondria become progressively less efficient, losing their functional integrity and releasing more oxygen molecules, thereby aggravating oxidative damage. Furthermore, dysfunctional mitochondria accumulate with aging [26]. In addition, ROS activate transcription factor NFκB, which induces the expression of a large variety of agents, including pro-inflammatory cytokines, such as IL-1/6 and TNF-α [27]. Consequently, by neutralizing ROS, adequate antioxidant defenses are essential to the homeostasis of both the cell and the entire organism.

## 5. The Human Eye

As mentioned above, the human eye is constantly exposed to sunlight and artificial light. Exogenous sources of ROS, such as UV light, visible light, ionizing radiations, chemotherapeutics, and environmental toxins, contribute to oxidative damage in the ocular tissues. UV rays, in addition to causing direct damage to the tissues, can also induce oxidative stress in the irradiated cells through the production of riboflavin ROS by activating triptophan and porphyrin, which can, in turn, activate cellular oxygen [28]. Oxidative stress is defined as an imbalance between ROS production and the cell’s antioxidant capacity, and long-term exposure to oxidative stress can lead to ocular disease.

In the aged eye, in the brain and, therefore, in neurodegenerative disease, normal antioxidant defense mechanisms decline, thereby increasing the vulnerability of the tissues to the effects of oxidative stress [29]. The surface of the eye and the cornea protect the other ocular tissues, and are highly exposed to oxidative stress of environmental origin [21]. Indeed, UV rays modulate the expression of antioxidants and pro-inflammatory mediators by interacting with the epithelial cells of the cornea [30]. H_2_O_2_ formation serves directly as a messenger for NF-kB activation [31]. The decline of antioxidant defenses in these tissues is manifested clinically by the onset of such pathologies as pterygium [32], corneal dystrophy [33], and Fuchs’ endothelial dystrophy [34]. The crystalline lens is highly sensitive to oxidative damage during aging, as its cells and their intracellular proteins are not replaced; this paves the way to cataractogenesis. H_2_O_2_ is the main oxidant involved in cataract formation and DNA damage to the lens and membrane pump systems, inducing loss of vitality of the epithelial cells and their death through necrotic and apoptotic mechanisms [35]. The TM, which is the anterior chamber tissue responsible for the drainage of aqueous humor, possesses numerous antioxidant defenses; nevertheless, it is particularly susceptible to mitochondrial oxidative damage, which strikes its endothelium, leading to malfunction and the increased intraocular pressure that signals the onset of glaucoma [36]. Moreover, a fairly significant correlation has been found between oxidative DNA damage in the human trabecular meshwork (HTM) and increased intraocular pressure and visual field defects in glaucoma patients [37]. Finally, the retina is particularly rich in dietary antioxidants, such as vitamin E, vitamin C, and macular carotenoids (lutein and zeaxanthin), which retard light-induced oxidative damage. These elements operate synergically; for example, vitamin E protects the membranes of the external segment of the DHA-rich photoreceptors, is regenerated by means of vitamin C, and is spatially distributed in complement with the carotenoids lutein and zeaxanthin [38]. Nevertheless, oxidative photo-stress can cause both acute and chronic damage to the retina. The pathogenesis of age-related macular degeneration involves oxidative stress and death of the retinal pigmented epithelium, followed by the death of the overlying photoreceptors. Consequently, as in the case of several neurodegenerative diseases, so also in the eye, the normal mechanisms of antioxidant defense decline with aging; this increases the vulnerability of both the eye and the brain to the deleterious effects of oxidative damage [29]. Free radicals of mitochondrial origin are thought to be among the main causes of mitochondrial DNA (mtDNA) damage. The generation of ROS induces damage to the I and III complexes and oxidation of the proteins in the mitochondria and cytoplasm, leading to mitochondrial dysfunction [39]. Several studies have found levels of 8-hydroxy-2′-deoxyguanosine (8-OHdG), a biomarker of oxidative DNA damage, in the mtDNA of the aged brain [40] and in the trabecular meshwork [41]. Moreover, high levels of 8-OHdG have been found in nuclear DNA (nDNA) and in mtDNA in post mortem brains of elderly subjects [42], and in trabecular mtDNA this damage has been seen to be greater in glaucoma patients than in healthy subjects. The greater sensitivity of mtDNA to oxidative damage may be due to a deficit in mtDNA repair mechanisms, lack of protection on the part of histone proteins, or the fact that the mtDNA is situated close to the internal mitochondrial membrane, where ROS are generated [42,43]. In this context, the use of dietary integrators that can oppose oxidative stress seems to be appropriate in the prophylaxis and/or therapy of degenerative diseases of the eye (Figure 2). 

## 6. The Properties of Polyunsaturated Fatty Acids

The ideal ratio between omega-6 and omega-3 ensures prevention against the harmful effects of an omega-6 overflow, which is caused by the production of prostanoids, leucotrienes, and harmful lipoxins.

The important extrinsic biological action of omega-3 fatty acids can be observed in a healthy individual through the structural and functional integrity of the cell walls (take-over in phospholipids), with particular reference to the nervous system, retina, kidneys, endocrine glands and gonads, and also through the local regulation of hormones (PGD, leukotrienes, thromboxanes) that exert an anti-inflammatory action, a systemic anti-degenerative action, and proper regulation of the immune system, of blood pressure and viscosity, and of vasoconstriction processes.

After ingestion, polyunsaturated fatty acids are distributed to cells and enriched in cellular membranes, where they influence cellular metabolism and survival. Polyunsaturated fatty acids are involved in various mitochondrial processes, including mitochondrial calcium homeostasis, gene expression, respiratory function, ROS production and mitochondrial apoptosis [44]. Furthermore, the polyunsaturated fatty acids are of particular interest, owing to their anti-inflammatory, antithrombotic, lipid-lowering, and vasodilating properties [45,46]. The omega-3 fatty acids EPA and DHA carry out various metabolic functions involving the whole organism (Table 1) [47], and their importance to the eye is being increasingly recognized. 

## 7. Dry-Eye Syndrome

**Figure 3:**
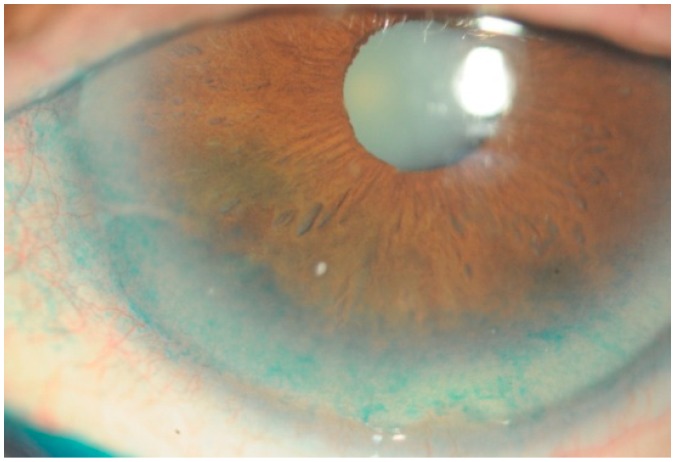
Dry-eye syndrome is a multifactorial disease of the ocular surface in which oxidative stress is greatly involved. Affected patents suffer from symptoms of discomfort and tear film instability, and visual quality is reduced proportionally to the severity of the disease. The Figure 3 shows an example of corneal epithelium injuries in a patient affected by dry-eye syndrome, as detected by means of Lissamine green staining. Diffuse corneal epithelium alterations appear as green dots in the frame-shift of the inferior cornea surface.

One of the most common diseases that affect the eye is dry keratoconjunctivitis; this is a multifactorial disorder and can occur as a result of numerous risk factors, such as, aging, autoimmune disease, menopause, the use of pharmaceutical drugs, and dysfunction of the cornea [49]. Dry eye is a disease of the ocular surface and involves tear film instability, symptoms of discomfort and visual disturbance (Figure 3). Tear film alterations and their subsequent effects on the corneal epithelium reduce visual quality, and in severe cases lead to significant visual impairment. There is a strong relationship between increased oxidative stress and the etiology of corneal epithelial alterations in blink-suppressed dry eyes [50]. This syndrome causes symptoms such as: a burning sensation, the sensation of a foreign body in the eye, lacrimation, and visual disorders. Increased osmolarity of the tear film and inflammation of the ocular surface occurs [51]. In dry-eye syndrome, the expression of antioxidant enzymes, such as superoxide dismutase, catalase, and glutathione peroxidase, is much lower than in controls, and correlates with the severity of dry-eye symptoms [52] (Figure 3). Supplementation with polyunsaturated fatty acids, in addition to reducing inflammation [53], can improve the lipid layer of the lacrimal film and normalize the function of the meibomian glands and lacrimal gland [54]. The utility of this type of dietary supplementation was asserted by two meta-analyses published in 2014. The first of these included nine articles published between 2002 in 2011 and reported the results recorded in 716 subjects [55]; the second included seven studies published between 2007 and 2013, and involved 790 subjects [56]. Both the burning sensation and reflex lacrimation improved [55], as did the Schirmer Test (which quantifies basal lacrimal secretion) and the lacrimal film rupture test [56] (which is regarded as an indicator of the stability of the lacrimal film). Rosacea patients with dry eye have also shown significant improvements in symptoms after dietary supplementation with O3FAs for 6 months [57]. Furthermore, a moderate daily dose of both forms of long-chain ω-3 EFAs, for three months, reduced tear osmolarity and increased tear stability in subjects with dry-eye disease [58]. Finally, the efficacy of dietary omega-3 fatty acids in alleviating dry-eye symptoms has been demonstrated in patients suffering from computer vision syndrome [59]. Recently, however, the “Dry Eye Assessment and Management Study Research Group” did not endorse the use of omega-3 supplements for patients with moderate-to-severe dry-eye disease [60].

## 8. Glaucoma

**Figure 4:**
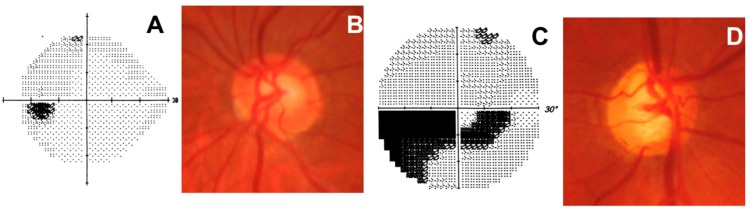
**Normal optic nerve** (left eye). (**A**) Normal visual field represented by the grayscale map. Darker areas indicate lower sensitivities, while higher sensitivities are represented by a lighter tone. This graphical representation allows easy interpretation of visual field loss, and is usually used to demonstrate vision changes to the patient; (**B**) Normal optic disc with a healthy neuroretinal rim; **Glaucomatous optic nerve** (right eye). (**C**) Dense glaucomatous arcuate scotoma; (**D**) Glaucomatous optic disc with evident narrowing of the superotemporal and inferotemporal neuroretinal rim.

Glaucoma is an optic neuropathy characterized by progressive atrophy of the retinal ganglion cells (RGC), which undergo apoptosis. The only therapy that has proved effective in glaucoma is lowering intraocular pressure. Interestingly, nutrition and intraocular pressure have been seen to have a clinical connection. Indeed, many studies have found a relationship between obesity and ocular hypertension [61,62], although the exact pathophysiology of elevated IOP in obesity remains unclear. Thus, although antioxidant supplementation cannot replace conventional therapy, it may be used as an adjunct to protect optic nerve head cells during glaucoma [63]. In addition to the RGC, the target tissues of this disease are the optic pathways, from the lateral geniculate nucleus to the visual cortex and, in the anterior segment, the trabecular meshwork (TM) (Figure 4). This latter is particularly sensitive to oxidative damage [22]. The aqueous humor is protected against free-radical and oxidant stress by a system that includes vitamins, antioxidant enzymes, chelating proteins, both lipid- and water-soluble antioxidants, and proteins such as albumins, which protect the TM [64]. The endothelial cells of the TM are constantly exposed to free radicals; in glaucoma, this becomes a fundamental pathogenetic factor. Indeed, a significant increase in 8-OH-dG has been observed in the TM cells [65], and this trabecular DNA damage has been correlated with increased intraocular pressure and visual field loss [66]. The cells of the TM are in contact with relatively high concentrations of H_2_O_2_ contained in the aqueous humor, and are susceptible to oxidative damage both in vivo and in vitro [67,68]. In the elderly, defenses against oxidative injury diminish, probably as a result of a reduction in the activity of superoxide dismutase (SOD), a key enzyme for the neutralization of H_2_O_2_ [69]. Many of the glaucomatous alterations of the TM structure are caused by the loss of endothelial cells [37]. Lipid peroxidation plays an important role in damage to the conventional outflow pathway [70]; indeed, the main targets of peroxidation reactions are proteins, cell membranes and nucleic acids (DNA and RNA), including mitochondrial DNA (mtDNA). Furthermore, mtDNA is less protected than nuclear DNA and is, therefore, more sensitive to free radical attack [22]. Thus, as a result of oxidative stress, glaucoma patients undergo trabecular mitochondrial deletions [26]. This leads to the functional decay of the endothelial cells of the TM and an alteration of their extracellular matrix, malfunctioning of the trabecular tissue, subclinical inflammation and changes in the extracellular matrix and cytoskeleton, and, finally, an alteration of TM motility [71]. Thus, miRNA expression could contribute to the malfunction of the outflow pathway in glaucoma [72]. miRNA expression is associated with cellular senescence in HTMcs [73], and senescent cells in the TM increase with aging and in POAG [74]. 

In addition to causing all these alterations, oxidative damage also produces an alteration of the proteome [75]. Indeed, the increase in oxidative stress involves the ubiquitin-proteasome system (UPS), with the consequent accumulation of damaged proteins [76], which obviously upsets cellular homeostasis. The proteins released in the anterior chamber then move posteriorly to activate first the glia and then apoptosis of the ganglion cells [41]. The anterior chamber, which contains the conventional outflow pathway of aqueous humor, is formed by the TM and Schlemm’s Canal. This pathway is basically made up of endothelial cells, which behave like those of a small vessel. In 2001, Wang discovered ELAM 1, a protein that is an early marker of atherosclerotic plaque, in the aqueous humor of glaucoma patients [77]. In 2012, Saccà et al. found all the other markers of atherosclerotic plaque which reflect the damage that occurs in anterior chamber endothelia, mainly including those of the trabecular meshwork, which is the main structure of this ocular segment that is injured by glaucoma [75]. Endothelial dysfunction occurs not only in normal-pressure glaucoma [78] but also in high-pressure glaucoma [71]. Like atherosclerosis, chronic endothelial dysfunction in glaucoma is characterized by an excess production of superoxide and endothelins and a decrease in the biosynthesis and/or bioavailability of nitric oxide [79]. Indeed, statins exert a protective action against glaucoma [80], particularly in the endothelial cells of the trabecular meshwork [81]. It is therefore intuitive that the use of polyunsaturated fatty acids in glaucoma has a physiopathological basis, given their beneficial effects on atherosclerosis and endothelial dysfunction [82].

## 9. Oxidative Damage and Aging

Cell senescence is the state of essentially irreversible cell-cycle arrest induced by a variety of stimuli, including oxidative stress [83]. Senescent cells display greater expression of genes that encode a range of secreted proteins, such as inflammatory cytokines, chemokines, and matrix-remodeling factors, which alter the local environment of the tissue and/or contribute to chronic inflammation [84]. The accumulation of senescent cells may contribute to the loss of tissue function in aging [85]. An age-related increase in the amount of ROS, a decline in the mechanisms of cellular repair or degradation, or both of these, leads to an increase in oxidized proteins and the consequent formation of amyloid [86], as occurs in Alzheimer’s disease, the accumulation of which can lead to oxidative stress and mitochondrial dysfunction [87]. In glaucoma, at the level of the traditional outflow pathway, which includes the TM and Schlemm’s canal, oxidative stress [68] contributes to increasing the “senescent cell” SA-β-Gal genotype [74]. Senescent cells are associated to a high level of intracellular ROS and display oxidative damage to DNA and proteins [88]. If intracellular oxidants increase, oxygen concentrations in the cellular environment are altered and antioxidant levels decline, thereby accelerating the onset of senescence. If, on the contrary, oxygen concentrations in the cellular environment are reduced, or ROS scavengers increase, senescence will be slowed [89,90]. Another factor that is involved in the promotion of early stress-induced senescence is TGF-β, which induces the activity of SA-β-Gal and increases the levels of microRNA (miRNA) of the senescence-associated genes [91]. This process is able to promote senescence in different cell types [92]. Therefore, senescent cells are associated with alterations in gene expression [93], protein processing [94] and metabolic processes [95] which, by overexpressing pro-inflammatory cytokines [96] and free radicals (ROS) can, in turn, induce apoptosis and reduce cellularity and change the ECM composition [74]. The expression of miRNA increases both with aging and in POAG [74] and contributes to the inefficient functioning of the outflow pathways in glaucoma [72,92]. Both cell senescence and oxidative damage are more marked in glaucoma patients. Interestingly, a diet high in saturated fat induces premature endothelial senescence [97], as dietary fat modulates oxidative stress in human endothelial cells. The so-called Mediterranean diet (MedDiet) protects these cells from oxidative stress, prevents cellular senescence and reduces cellular apoptosis [98]. Indeed, Tourtas et al. [99] found that omega-3 and -6 fatty acids contribute to suppressing the formation of H_2_O_2_ mediated by the NfκB pathway, which is dependent on the degree of oxidative stress [55]. Moreover, the polyunsaturated fatty acids, particularly the omega-3 PUFA, also have an effect on the TM matrix, preventing the accumulation of H_2_O_2_ [99]. In other words, this kind of supplementation seems, at least in vitro, to exert a prophylactic effect on glaucomatous disease. In addition, in rats this diet has been seen to reduce glial cell activation induced by increased intraocular pressure [100].

## 10. Age-Related Macular Degeneration

**Figure 5:**
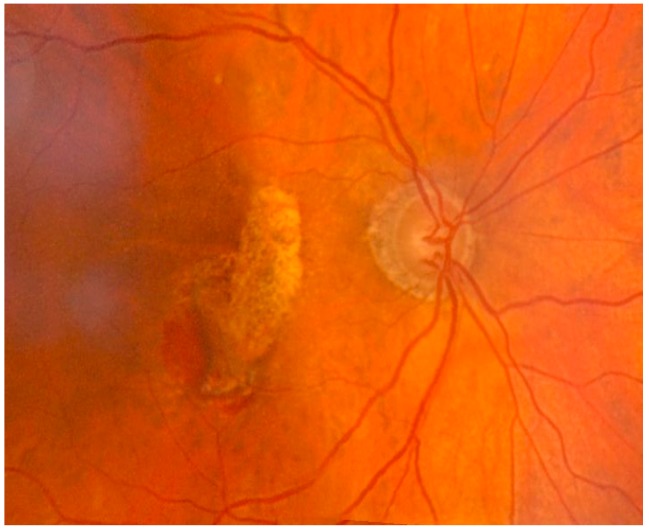
Exudative age-related macular degeneration with parafoveal neovascular component localized inferiorly in the area of intraretinal hemorrhage. The area of atrophy of the retinal pigment epithelium in the macular and superior paramacular sites. The exact pathological mechanisms leading to the different forms of AMD are still unclear. Oxidative stress and inflammation seem to play a major role in both AMD and aging, leading to the formation of abnormal extracellular matrix. This event results in altered behavior of RPE choriocapillaries, ultimately leading to atrophy of the retina, RPE, and choriocapillaries, which is paralleled by neovascularization of the choroid.

In proportional terms, the part of the body in which most oxygen is consumed is the retina. Visible light reacts with the chromophores, giving rise to final products that are able to increase ROS production and oxidative damage. Moreover, the retina is highly sensitive to this type of damage, as it contains high levels of polyunsaturated fatty acids, which can easily become oxidized. From a physiological standpoint, omega-3 long-chain polyunsaturated fatty acids in the retina exert a protective function against light, ischemia, oxygen, inflammation, and age-associated pathologies of the vascular and neural components of the retina [101] (Figure 5). The pathological bases of age-related macular degeneration (AMD) are to be found in oxidative stress. During aging, the antioxidant capacity diminishes and the efficiency of repair systems is compromised. Indeed, oxygen levels are highest in the choroid, but decline significantly across the outermost retina, creating a steep oxygen gradient towards the retina and internal segments of the photoreceptors, which contain high levels of polyunsaturated fatty acids [102]. The result is retinal dysfunction. This is characterized by an accumulation of liposomial lipofuscin in the cells of the pigmented epithelium (RPE) and the presence of drusen, i.e., extracellular deposits between the basal lamina of the RPE and the internal collagen layer of Bruch’s membrane (Figure 5). The degeneration and cell death of the cells of the RPE cause secondary negative effects on the neural retina, leading to vision loss. 

The omega-3 (Ω-3) fatty acids, or PUFA n-3, are made up of linolenic acid, docosahexaenoic acid (DHA) and eicosapentaenoic acid (EPA). EPA and DHA are the main representatives of the Ω-3 fatty acids. They belong to the category of essential fatty acids (which also includes omega-6 fatty acids, known to play a role in maintaining the integrity of cell membranes). These fatty acids are able to control and resolve inflammation [103,104,105]. Moreover, high levels of DHA are found in the retina, specifically in the discs of the external segment of the rods, suggesting that DHA plays an essential functional role in the retina [101,106]. Indeed, DHA influences the biophysical properties of the membrane, is a central regulator of the visual cycle [107], controls the membrane transport systems, and is a precursor to the synthesis of other active molecules. Thus, DHA may play a protective role in degenerative diseases of the retina, particularly in AMD [108]. EPA regulates the metabolism of lipoproteins and suppresses the expression of various inflammatory compounds that can damage the extracellular matrix, including Bruch’s membrane, and leads to the neovascularization seen in AMD [109,110]. Given the crucial role of inflammation in the pathogenesis of AMD, EPA may be a protective factor in preventing, or at least slowing down, the progression of the disease [108]. Indeed, DHA protects the photoreceptors against the apoptosis induced by oxidative stress and promotes their differentiation in vitro, by activating the receptor X of the retinoid (RXR) and the ERK/MAPK pathway, thereby regulating the expression of anti- and pro-apoptotic proteins [111]. In animal models, dietary supplementation with antioxidants and omega-3 fatty acids has been seen to rapidly modify the fatty acid content and to increase the retinal content of EPA by over 70%, without influencing the content of rhodopsin, thereby protecting the retina against light-induced oxidative stress [112]. In a meta-analysis of nine studies, which provided data on a sample of 88,974 subjects, Chong et al. analyzed the effects of a diet rich in omega-3 fatty acids and the consumption of fish in the primary prevention of age-related macular degeneration; they concluded that the consumption of these fatty acids was associated with a lower risk of AMD [110].

## 11. Conclusions

This article has some limitations, since we took into consideration only those papers that dealt principally with ocular diseases and their pathogenesis. We, therefore, selected those articles that focused on the homeostatic cellular mechanisms of the polyunsaturated fatty acids that were able to interfere with the pathogenetic mechanisms of the ocular diseases that we considered. Our review is, therefore, limited to suggesting the possible interactions between polyunsaturated fatty acids and ocular diseases that are underpinned by oxidative damage, mitochondrial dysfunction, and inflammation. Moreover, we tried to correlate the causes of these diseases with the pharmacological actions of these molecules. Finally, we know that many aspects of the interactions between polyunsaturated fatty acids and ocular tissues are worthy of further investigation and are far from having definitive answers.

Systemic increases in reactive oxygen species, and their association with inflammation, have been proposed as an underlying mechanism linking degenerative diseases of the eye. The prevention of these highly-invalidating conditions is of considerable epidemiological importance and must take into account the mutagenic mechanism of these pathologies. 

The contribution of alimentary antioxidants and anti-mutagens is very important in maintaining the ocular tissues and preventing these diseases. In glaucoma and macular degeneration, as well as in dry-eye syndrome, pharmacological therapy and chemoprophylaxis already include the administration of antioxidants and preventive agents in order to attenuate ocular damage.

In this scenario, we can assert that dietary supplementation with polyunsaturated fatty acids is particularly indicated for the health of the eyes.

Further research should be directed toward remedying the clear lack of studies that take into account the biochemical, immunological, and genetic aspects of eye diseases together with nutritional aspects, in order to identify specific molecular mechanisms involved in the positive effects of the consumption of polyunsaturated fatty acids and, in particular, those molecular mechanisms that can yield benefits in the adjuvant therapy of eye diseases.

## Figures and Tables

**Table 1:** Omega-3 EPA and DHA have many features and are necessary in health and in disease states [48].

Functions of Omega-3 EPA and DHA	
Biological	Clinical
They exert an anti-aggregant activity; reduce neuroinflammation	They reduce the risk of coronary artery disease and sudden death;
Increase HDL and lower LDL;	are essential to fetal development;
Modulate the endogenous synthesis of prostaglandins and leukotrienes, exerting an anti-inflammatory action, especially in rheumatoid arthritis and psoriasis;	improve some forms of depression; efficacious in attention deficit syndrome;
Favor the equilibrium of the immune system, especially in lupus and Crohn’s disease; modify physical properties of membrane lipids.	help in the treatment of schizophrenia and bipolar disorders.
